# EVALUATING A TRANSITIONAL CARE PROGRAM FOR OLDER ADULTS WITH FRAILTY BETWEEN HOSPITAL AND HOME

**DOI:** 10.1093/geroni/igad104.2792

**Published:** 2023-12-21

**Authors:** Ji Yeon Lee, Eunhee Cho, Sue Kim, Gwang Suk Kim, Kyung Hee Lee, Chang Oh Kim

**Affiliations:** Yonsei University, Seoul, Republic of Korea; Yonsei University College of Nursing, Seoul, Republic of Korea; Yonsei University, Seoul, Republic of Korea; Yonsei University College of Nursing, Seoul, Republic of Korea; Yonsei University, Seoul, Republic of Korea; Yonsei University, Seoul, Republic of Korea

## Abstract

Older adults with frailty experience numerous health problems even after hospital discharge, highlighting the necessity of continued intervention until their recovery and return to daily routines. This mixed methods research (i.e., quantitative and qualitative studies) aimed to evaluate the effectiveness of a transitional care program developed for older adults with frailty during the transition from hospital to home and how it was helpful. From June to December 2021, 32 older adults with frailty were enrolled in a randomized controlled trial at a university-affiliated hospital in Korea. They were allocated to either an intervention group (n=16) or a control group (n=16) according to a random number sequence. The intervention group received a 12-week transitional care program that included inpatient care, structured discharge, and follow-up interventions (e.g., home visits and phone follow-ups); the control group received usual care only. At 12 weeks, program participants (eight frail older adults and eight family caregivers) had interviews for the qualitative research. The results showed that the intervention group had a higher score in knowledge readiness (p=.046) at discharge, empowerment (p=.032), and connectedness at four weeks (p=.037); community resource utilization (p <.001) and emergency room visit rate (p=.049) at 12 weeks; and improvement in depression (p <.001), family interaction (p=.045), and subjective health status (p=.002) over time. Participants described the program as a supportive, connected, timely, and personalized intervention. Overall, the study demonstrated that the transitional care program effectively improved the health outcomes of older adults with frailty and facilitated a safe transition between hospital and home.

